# Induction of labor is not associated with decreased rates of breastfeeding in late preterm pregnancies

**DOI:** 10.1016/j.xagr.2026.100637

**Published:** 2026-03-28

**Authors:** Alice Sutton, Daniella Rogerson, Samantha Thomson, Gina Frugoni, Cynthia Gyamfi-Bannerman

**Affiliations:** 1 Department of Obstetrics, Gynecology and Reproductive Sciences, University of California San Diego, La Jolla, CA; 2 Division of Maternal-Fetal Medicine, Department of Obstetrics, Gynecology and Reproductive Sciences, University of California San Diego, La Jolla, CA

**Keywords:** breastfeeding complications, breastfeeding rates, cesarean delivery, chestfeeding, government payor insurance, lactation, late preterm delivery, late preterm induction, medically indicated induction of labor, private insurance

## Abstract

**Background:**

Some studies suggest that induction of labor at term is associated with lower rates of breastfeeding than spontaneous labor.

**Objective:**

Our objective was to evaluate whether late preterm medically indicated induction of labor is associated with decreased rates of breastfeeding and/or increased rates of breastfeeding complications at the time of discharge from the delivery hospitalization.

**Study Design:**

This secondary analysis of a randomized trial of individuals at high risk for late preterm delivery, defined as delivery between 34+0 and 36+6 weeks, included nonanomalous, singleton pregnancies and excluded those with unlabored cesareans or preterm prelabor rupture of membranes. The parent study collected detailed data on breastfeeding and the presence of breastfeeding difficulties, defined as issues in milk production or infant feeding. Subjects with incomplete breastfeeding data were additionally excluded. Participants undergoing late preterm indicted inductions were compared to those who presented with spontaneous preterm labor. The primary outcome, the rate of breastfeeding, was compared between groups. Breastfeeding difficulties were also compared. Baseline demographics were compared using bivariable analyses. We fit logistic regression models to adjust for confounders related to breastfeeding.

**Results:**

Two thousand one hundred thirty participants were included. Spontaneous and induction groups were similar in age, tobacco use, gestational diabetes, and insurance type but the induced group had higher body mass index, rates of chronic hypertension and hypertensive disorders of pregnancy (HDP). The rate of breastfeeding did not differ in induced vs spontaneous participants (69.0% vs 68.7%, *P*=.90). However, breastfeeding difficulties were more common in the induced group in unadjusted analyses (38.8% vs 32.3%, *P=*.01). After adjusting for confounders, neither breastfeeding rates nor breastfeeding difficulties were different between groups. Of note, government insurance was an independent risk factor for low breastfeeding rates and induction was associated with a decreased rate of cesarean delivery

**Conclusion:**

Medically indicated late preterm labor induction was not associated with decreased rates of breastfeeding or increased breastfeeding problems at time of discharge from the delivery hospitalization. Those with government funded insurance may need additional breastfeeding support.

**AJOG Global Reports at a Glance**

**Why was this study conducted?**

To examine whether medically indicated late preterm induction of labor is associated with decreased rates of breastfeeding or increased rates of breastfeeding problems when compared with spontaneous late preterm deliveries.

**Key findings?**

Medically indicated late preterm induction of labor was not associated with decreased breastfeeding, nor increased incidence of breastfeeding problems, at the time of discharge from admission for delivery.

**What does this add to what is known?**

This study suggests that the breastfeeding challenges associated with medically indicated late preterm delivery are not caused by the induction process.

## Introduction

Breastfeeding has numerous maternal and neonatal benefits, and the World Health Organization, the American College of Obstetricians and Gynecologists, and the American Academy of Pediatrics recommend exclusive breastfeeding for the first 6 months of life.^1^ Unfortunately, late preterm infants are breastfed at lower rates than term infants,^1^ although late preterm infants may experience more significant benefits from human breastmilk than their term counterparts.^2^ Late preterm infants are at risk for breastfeeding complications due to a variety of infant and parental factors including infant immaturity, impaired milk emptying, delayed lactogenesis, and separation of the parental/neonatal dyad.^1^

Given the benefits of human breast milk, researchers have attempted to elucidate which peripartum medical interventions may impact breastfeeding. Some studies have demonstrated that induction of labor with synthetic oxytocin may be associated with decreased breastfeeding in term populations.^3^ It is unknown how induction of labor is associated with breastfeeding in a late preterm population. The number of late preterm deliveries in the United States has increased in part due to medically indicated preterm inductions.^4^ This represents a growing population at risk for low breastfeeding rates despite the known benefits in this population. Better understanding of the risk factors for decreased rates of breastfeeding in the late preterm population would facilitate patient counseling and targeted interventions to support breastfeeding.

While some evidence suggests induction of labor is a risk factor for breastfeeding difficulties at term, it is unknown if this association persists in a late preterm population. Our objective was to determine whether labor induction affects breastfeeding and/or breastfeeding difficulties in populations at risk for late preterm delivery. We hypothesized that induction of labor in a late preterm population would be associated with decreased rates of breastfeeding compared with infants born after spontaneous late preterm labor.

## Materials and methods

This is a secondary analysis of the ALPS study (Antepartum Late Preterm Steroids) which was conducted within 17 university-based hospitals within the *Eunice Kennedy Shriver* National Institute of Child Health and Human Development Maternal-Fetal Medicine Units Network between 2010 and 2015.^5^ In the parent trial, patients with a live, singleton, pregnancy at elevated risk of delivery between 34 weeks 0 days to 36 weeks 6 days due to preterm labor, spontaneous rupture of membranes, or medically indicated delivery were randomized to receive either an antepartum course of betamethasone or a placebo. Participants undergoing indicated preterm induction of labor (IOL) were included if the IOL was anticipated to start by 36 weeks 5 days; among the exclusion criteria for the parent trial was an exclusion for individuals with unclear dating. Abstracted study data included a series of questions about breastfeeding and the presence of breastfeeding difficulties. Other study procedures were previously detailed.^5^ Because this was a secondary analysis of publicly deidentified data, our IRB deemed this study to be exempt.

For this secondary analysis, we included all persons who presented in preterm labor and those who had a medical indication for delivery who underwent induction of labor. We excluded participants who underwent unlabored cesarean delivery. We also excluded participants with preterm prelabor rupture of membranes (PPROM) or who required induction for PPROM, because we considered the neuro-endocrinologic state of participants with ruptured membranes to be different from individuals without labor. We additionally excluded those with major congenital anomalies, and those with incomplete data in 1 of 2 areas: (1) breastfeeding and breastfeeding issues, or (2) indication for induction. All individuals with a recorded indication for induction, including “other” or “elective,” were included in the induced group. The noninduced group was comprised of all individuals without an indication for induction and excluding those who had an elective cesarean delivery.

We reviewed maternal demographics including insurance type, maternal comorbidities, gestational age at randomization and delivery, whether labor was induced, whether breastfeeding was initiated, and the presence of breastfeeding problems. Payor status was defined as self-pay or uninsured, private insurance, or government assisted insurance. Government assisted insurance included Medicaid or any state funded program.

The primary outcome was the rate of breastfeeding, defined as any attempt at breastfeeding prior to infant discharge. The secondary outcome was the presence of breastfeeding difficulties, defined as the presence of any of the following: trouble sucking or latching, choking, sleepy infant, disinterested infant, distracted infant, nursed too often, delayed milk production, insufficient milk supply, breasts leaked too much, infant gained/lost too much weight, nipples sore/cracked/bleeding, breast engorgement, breast yeast infection, clogged milk duct, breast infection/abscess, cholestasis, and “other.” The presence of breastfeeding difficulties was abstracted from the medical chart by a trained study nurse. Other secondary outcomes included various maternal and neonatal outcomes that may affect breastfeeding, including mode of delivery, infant sex and low 5 minute APGAR (defined as <7).

Baseline demographics and maternal characteristics were compared between the induced group vs the noninduced group. Continuous variables were compared using Student’s t-test or Wilcoxon Rank Sum, for normally distributed or nonparametric variables, respectively. Categorical variables were compared using Chi square or Fisher’s exact test, as appropriate. We then compared the primary and secondary outcomes between groups and estimated the association between the outcome and the exposure (induction) by fitting logistic regression models. Confounders were defined by those characteristics that were either clinically significant or statistically different to a *P*-value of .2. Significance for the outcomes was defined as a *P*-value of .05. All analyses were on SAS version 9.4.

## Results

**Figure 1:**
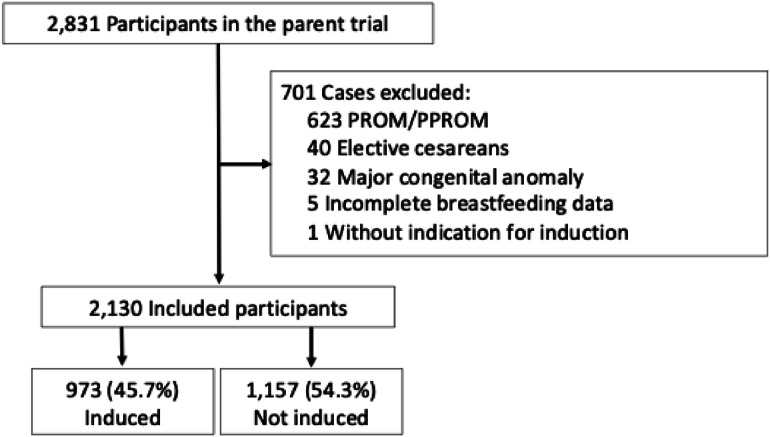
Flowchart of study population

Of the 2,831 participants in the parent trial, 623 (22%) were excluded for PROM/PPROM, 40 (1.4%), were excluded for elective cesarean, 32 (1.1%) were excluded for major anomalies, 5 (0.2%) for incomplete breastfeeding data and 1 (0.04%) for lacking an indication for induction (Figure 1). Of the 2,130 individuals included in the current analysis, 973 (45.7%) were induced and 1157 (54.3%) were not induced. Baseline demographics and other characteristics are listed in Table 1. The induced group had significantly more nulliparous participants, a higher median BMI, and was more likely to have chronic hypertension. The mean gestational age at delivery in the induced group was 36w1d compared to 36w3d in the noninduced group (*P*<.001). Betamethasone exposure was similar in the induced vs noninduced groups (Table 1).

Sutton. Induction of labor is not associated with decreased rates of breastfeeding in late preterm pregnancies. Am J Obstet Gynecol 2026.

**Table 1:** Demographics and baseline characteristics

	Not induced n=1157	Induced n=973	*P*
Mean age in years^a^	28.2±5.98	28.2±6.02	.88
Advanced maternal age	199 (17.6)	161 (16.9)	.68
Median BMI^b^	25.1 (21.8–30.3)	27.4 (22.9–34.1)	<.001
Nulliparous	233 (20.1)	387 (39.8)	<.001
Primary source of medical payment			.37
*Self-pay*	41 (3.5)	39 (4)	
*Private*	390 (33.7)	352 (36.2)	
*Government-assisted*	726 (62.7)	582 (60)	
Patient smoked cigarettes at any time during pregnancy	171 (14.8)	129 (13.3)	.31
Gestational diabetes	105 (9.1)	131 (13.5)	.001
Chronic hypertension	86 (7.4)	185 (19)	<.001
Hypertensive disorders of pregnancy			<.001
*None*	955 (82.5)	280 (28.8)	
*Gestational hypertension*	53 (4.6)	77 (7.9)	
*Preeclampsia, preeclampsia with severe features, eclampsia, or HELLP*	149 (12.9)	616 (63.3)	
Gestational age at delivery in weeks^a^	36w3d ±1d	36w1d±1d	<.001
Betamethasone exposure	588 (50.8)	492 (50.6)	.91

Data are n (%) unless otherwise specified.

*HELLP*, Hemolysis, elevated liver enzymes, low platelets.

^a^Mean ± standard deviation.

^b^Median (95% confidence interval).

Primary, secondary, and other selected outcomes are listed in Table 2. Breastfeeding was initiated at similar rates in both groups with 69.0% in the induced group and 68.7% of the noninduced group (*P*=.90). Adjusted odds ratios for breastfeeding outcomes are listed in Table 3. After adjusting for confounders that differed at baseline, including nulliparity, hypertension, payor status, and BMI, there remained no association between late preterm induction and breastfeeding rates (OR 0.98, 95% CI 0.80–1.20, *P*=.84). Breastfeeding problems were present in 38.8% of the induced group and 32.3% of the noninduced group (*P*=.001), however, after adjusting for the same baseline confounders this difference was not significant (OR 1.17, 95% CI 0.93–1.48, *P*=.19). The average gestational age at delivery for breastfed and nonbreastfed infants was similar, delivering at 36w2d±1d and 36w2d±1d (*P*=.074), respectively. Of note, individuals with government-assisted insurance were found to be less likely to breastfeed when compared to individuals with private insurance (OR 0.28, 95% CI 0.15–0.53, *P*<.001).

**Table 2:** Outcomes for induced and not induced participants

	Not induced n=1157	Induced n=973	*P*
*Primary and secondary outcomes*			
Breastfeeding initiated	795 (68.7)	671 (69)	.90
Breastfeeding problems	256 (32.3)	260 (38.8)	.001
*Other selected outcomes*			
Cesarean delivery	495 (42.3)	266 (27.3)	<.001
Infant sex			.53
Male	607 (52.5)	497 (51.1)	
Female	550 (47.5)	476 (48.9)	
Low Apgar	35 (3.1)	34 (3.6)	.55
Parent study primary outcome^a^	145 (12.5)	130 (13.4)	.57
Major respiratory morbidity^b^	119 (10.3)	92 (9.5)	.52
NICU admission	416 (36.0)	436 (44.8)	<.001

Data are n (%) unless otherwise specified. Breastfeeding = breastfeeding attempted before discharge from hospital. Low Apgar = 5-minute Apgar <6.

^a^Parent Study Primary Outcome = composite of respiratory morbidity occurring by the first 72 hours of life including continuous positive airway pressure (CPAP) or high flow nasal cannula (HFNC) for ≥2 hours, oxygen (O2) requirement with FiO2 of ≥30% for ≥4 hours, any mechanical ventilation or perinatal death.

^b^Major Respiratory Morbidity = O2 requirement ≥24 hours of life and CPAP/HFNC ≥12 hours of life.

**Table 3:** Adjusted odds ratios for breastfeeding outcomes in induced and not induced participants

	Unadjusted OR (95% CI)	Adjusted OR (95% CI)	*P^a^*
Breastfeeding initiated	1.01 (0.84–1.22)	0.98 (0.80–1.20)	.84
Breastfeeding problems	1.33 (1.07–1.65)	1.17 (0.93–1.48)	.19

^a^Adjusted for nulliparity, hypertension, gestational diabetes, payor status, maternal age, and BMI.

Other secondary outcomes included the rate of cesarean, which was 27.3% vs 42.3% in the induced and noninduced groups respectively (*P*<.001). The lower rate of cesarean in the induced group persisted even after adjusting for confounders (OR 0.36, 95% CI 0.29–0.44, *P*<.001).

## Comment

### Principal findings

In this secondary analysis we investigated the association between labor induction and breastfeeding initiation in a population at risk for late preterm delivery and found no association between labor induction and breastfeeding initiation. There was also no association between labor induction and breastfeeding problems after adjusting for confounders.

### Results in the context of what is known

These findings shed light on an understudied area of breastfeeding medicine as most studies on the effect of induction of labor on breastfeeding exclude individuals who delivered prematurely. A 2017 review article on breastfeeding outcomes after oxytocin use in childbirth identified 26 studies, only one of which included any late preterm deliveries.^3^^,^^6^, ^7^, ^8^, ^9^, ^10^, ^11^ Late preterm infants may particularly benefit from breastmilk, despite facing increased feeding challenges.^12^ Given that 7.69% of deliveries in the United States were late preterm in 2024, this represents a significant number of parental/infant dyads at risk for breastfeeding complications.^13^

### Clinical implications

We intentionally took a pragmatic approach to examine the impact of late preterm induction of labor on breastfeeding so that our findings would be clinically relevant. In current obstetric practice, elective late preterm inductions of labor are not permitted, so all patients were being induced for medical indications. Despite the medical challenges these dyads faced, and despite going through the labor induction process, breastfeeding was initiated at similar rates and breastfeeding problems were encountered at similar rates to their peers who labored spontaneously in the late preterm period. This information may be helpful to obstetricians, midwives, lactation consultants and others who care for these patients. Although lactation support is resource-intensive, our data suggest that these medically complex patients remain appropriate candidates for breastfeeding interventions. Moreover, patients undergoing medically indicated late-preterm induction of labor may be reassured and encouraged by the observation that their breastfeeding outcomes do not differ from those of noninduced peers who delivered in the late preterm period.

### Research implications

Our data did suggest that government-assisted insurance may be associated with lower rates of breastfeeding. Given the association between social determinants of health and breastfeeding, this finding is both plausible and also suggestive of future avenues for research and even intervention.^14^ We also noted a decreased cesarean delivery rate in the induced group compared to noninduced participants, even after excluding those with preterm rupture of membranes. There have been few opportunities to examine the effect of late preterm induction on rate of cesarean section, and this represents a novel finding warranting further exploration.

### Strengths and limitations

In contrast to previous studies that focused on a single induction agent,^3^ a strength of our study is its pragmatic approach to evaluating the total impact of labor induction processes. Labor induction is more than the administration of synthetic oxytocin and cervical ripening agents; it impacts diverse aspects of the labor experience including modalities for pain control, length of stay, and nursing care. This pragmatic approach better equips clinicians to anticipate which patients may experience breastfeeding challenges and is more useful for patient counseling. Additional strengths of this study include rigorous and granular data collection in the original randomized controlled trial, and complete indications for induction of labor.

Limitations of this study include that it is a secondary analysis, and thus we were limited by the variables collected in the parent trial. While robust data on the initiation of breastfeeding were collected, potential nuances such as rates of breastfeeding weeks to months postpartum, and rates of exclusive breastfeeding, were not collected. We also lacked data on the dose and duration of synthetic oxytocin, and we were unable to control for exposure to neuraxial anesthesia. Breastfeeding at discharge from the delivery hospitalization is an early step in the breastfeeding relationship; we acknowledge that other measures including presence of exclusive breastfeeding at 6 or 12 months have been suggested as more meaningful indicators of population health. However, this is one of the first studies to examine late preterm induction of labor and breastfeeding and future work can build upon this foundation.

## Conclusions

Our study suggests that late preterm induction of labor is not associated with decreased breastfeeding rates, nor increased breastfeeding problems, after adjusting for confounders. This provides additional context for patient counseling, which is needed as the mixed data regarding the effect of induction of labor on breastfeeding has already been reported in the lay press^15^^,^^16^ and widely repeated on the internet. We also found that government payor insurance was associated with decreased breastfeeding rates, which may help direct resources to the population(s) that would benefit the most from additional support.

## Data availability statement

The authors confirm that data supporting the findings of this study are available within the article.

## Funding statement

This work received no specific grant from any funding agency.

## Ethics approval statement

The parent trial research protocol was approved by the Institutional Review Board of each clinical site where the randomized control study took place. This secondary analysis was exempt from review given the deidentified nature of the data used for secondary analysis.

## Patient consent statement

All patients in the parent trial provided voluntary written informed consent prior to study participation and only deidentified data was reviewed in this secondary analysis.

## CRediT authorship contribution statement

**Alice Sutton:** Writing – review & editing, Writing – original draft, Project administration, Investigation, Conceptualization. **Daniella Rogerson:** Writing – review & editing, Visualization. **Samantha Thomson:** Writing – review & editing. **Gina Frugoni:** Writing – review & editing. **Cynthia Gyamfi-Bannerman:** Writing – review & editing, Supervision, Resources, Methodology, Investigation, Formal analysis, Conceptualization.

## Declaration of competing interest

The authors report no conflict of interest.
